# Compositional and Functional Disparities in the Breast Oncobiome Between Patients Living in Urban or Rural Areas

**DOI:** 10.3390/genes16070806

**Published:** 2025-07-09

**Authors:** Fazia Ait Zenati, Simone Baldi, Leandro Di Gloria, Ferhat Djoudi, Sara Bertorello, Matteo Ramazzotti, Elena Niccolai, Amedeo Amedei

**Affiliations:** 1 Laboratory of Microbial Ecology, Department of Microbiology, University of Bejaia, Bejaia 06000, Algeria; fazia.aitzenati@univ-bejaia.dz (F.A.Z.); ferhat.djoudi@univ-bejaia.dz (F.D.); 2 Department of Experimental and Clinical Medicine, University of Florence, 50134 Florence, Italy; simone.baldi@unifi.it (S.B.); sara.bertorello@unifi.it (S.B.); elena.niccolai@unifi.it (E.N.); 3 Department of Biomedical, Experimental and Clinical Sciences “Mario Serio”, University of Florence, 50134 Florence, Italy; leandro.digloria@gmail.com (L.D.G.); matteo.ramazzotti@unifi.it (M.R.); 4 Network of Immunity in Infection, Malignancy and Autoimmunity (NIIMA), Universal Scientific Education and Research Network (USERN), 50139 Florence, Italy

**Keywords:** breast cancer, oncobiome, breast microbiota, 16S Sequencing, living area

## Abstract

**Background/Objectives**: Breast cancer (BC) is the leading cause of cancer incidence and mortality among women and the recent identification of a resident mammary microbiota has highlighted its potential role in breast carcinogenesis. Given that environmental and socioeconomic factors influence both BC prevalence and tumor-associated bacterial composition, this study aimed to evaluate the compositional and functional features of the mammary microbiota in cancerous (oncobiome) and adjacent healthy BC tissues from patients living in urban and rural areas. **Methods**: Microbiota composition in both the oncobiome and adjacent healthy BC tissues was analyzed using 16S rRNA sequencing. **Results**: Significant variations in breast oncobiome composition were observed among BC patients from urban and rural areas. A statistically significant β dispersion among breast oncobiome of patients from urban or rural areas was highlighted. Specifically, the genera *Selenomonas*, *Centipeda*, *Leptotrichia*, *Neisseria* and *Porphyromonas* were found exclusively in BC tissues of patients from rural areas. Additionally, bacteria from the Neisseriaceae, Porphyromonadaceae, and Selenomonadaceae families, as well as the *Selenomonas* genus, were significantly enriched in the oncobiome of rural BC patients. Furthermore, the results of the PICRUSt2 (phylogenetic investigation of communities by reconstruction of unobserved states) revealed a significant increase in phospholipid biosynthesis pathways in breast oncobiome of patients from rural areas compared to those from urban areas. **Conclusions**: This study provides evidence of distinct compositional and functional differences in the breast oncobiome between BC patients from rural and urban areas. These findings suggest that environmental factors influence local microbiome composition, potentially contributing to BC development and/or progression.

## 1. Introduction

Breast cancer (BC) is the leading cause of cancer-related mortality in over 100 countries, ranking first in both incidence and death rates among women worldwide. It is the most frequently diagnosed cancer in females, accounting for 11.7% of all cases, and currently stands as the fifth leading cause of cancer-related deaths globally, with an estimated 685,000 fatalities [1]. In recent years, BC incidence rates in emerging countries such as those in South America, Africa, and Asia have risen to levels comparable to those in Western countries, reducing international disparities in BC morbidity [2]. This increase is attributed to a higher prevalence of reproductive and hormonal risk factors, including early menarche, late menopause, delayed childbirth, fewer children, reduced breastfeeding, use of menopausal hormone therapy or oral contraceptives, along with lifestyle westernization and expanded access to organized or opportunistic mammography screening [1,3].

In Algeria, for example, BC remains a significant health concern. According to a 2018 report, 11,847 new cases were reported, accounting for 24% of all cancer diagnoses in the country. The median age at diagnosis was 48 years, with 66% of cases occurring in women under 50 [3,4]. However, comprehensive data on disease severity remain scarce, and current evidence indicates regional variations in BC incidence, with a substantial number of BC-related deaths going unreported, mainly due to inadequate screening and late-stage diagnosis, especially in rural areas [5]. In recent years, research has increasingly focused on the human microbiome and its role in cancer development. The discovery of a resident mammary microbiota has led to growing interest in the potential role of the breast oncobiome in BC carcinogenesis. Specifically, microbial communities may influence BC risk through several mechanisms, including the following: (i) induction of inflammation, (ii) alterations in the tissue microenvironment, and (iii) metabolic or direct toxic effects [6]. Preliminary studies suggest that the microbial composition of histologically normal breast tissue obtained under sterile conditions differs from that of cancerous tissue [7]. For instance, Esposito and colleagues found significant differences in microbial taxa abundance between breast tumor tissues and adjacent healthy tissues [8]. Moreover, a recent revision of The Cancer Genome Atlas database confirmed the presence of bacteria in both normal and cancerous breast tissues, with Proteobacteria, Actinobacteria, and Firmicutes being the predominant phyla [9]. Finally, we identified a sexually dimorphic breast-associated microbiome, termed the ‘breast microgenderome’, where dysbiosis affects the entire breast tissue in females, but remains localized to the tumor site in males [10].

Usually, BC tissues exhibit lower bacterial abundance than adjacent healthy tissues, along with reduced expression of antimicrobial response gene [11]. Notably, numerous studies have demonstrated that chronic inflammation often precedes cancer development, suggesting a potential bacterial “field effect” in promoting neoplastic processes. Additionally, healthy tissues surrounding tumors frequently undergo changes due to immune cell infiltration, fibrosis, and tumor-associated inflammation [12]. Consequently, alterations in the normal breast microbiota may contribute to inflammatory responses that influence carcinogenesis.

Given that environmental and socioeconomic factors impact both BC prevalence and tumor-associated microbiota composition, this exploratory study aims to characterize and compare the composition and function of the mammary microbiota in cancerous and adjacent healthy breast tissues from patients living in urban and rural areas.

## 2. Materials and Methods

### 2.1. Patient Enrolment

Tumors and adjacent healthy breast tissues were obtained from patients with diagnosed BC attending at Khelil Amrane University Hospital of Bejaia from January to October 2022. Patients who had taken antibiotics, probiotics, prebiotics, and symbiotics were excluded from this study. Tissue samples were immediately frozen at −80 °C to prevent environmental contamination.

### 2.2. Characterization of Breast Tissue Microbiota

Genomic DNA was extracted using a DNeasy PowerSoil Pro Kit (Qiagen, Hilden, Germany) from frozen (−80 °C) cancerous and healthy adjacent tissues, according to the manufacturer’s instructions. Briefly, 0.25 g of tissue was added to a bead-beating tube and homogenized with TissueLyser LT (Qiagen, Hilden, Germany) for 5 min at 50 Hz. Afterwards, DNA was captured on a silica membrane in a spin column format, washed, and eluted. The quality and quantity of the extracted DNA were assessed with both a NanoDrop ND-1000 (Thermo Fisher Scientific, Waltham, MA, USA) and Qubit Fluorometer (Thermo Fisher Scientific, Waltham, MA, USA), and then the DNA were frozen at −20 °C.

Subsequently, total DNA samples were sent to IGA Technology Services (Udine, Italy) where amplicons of the variable V3–V4 region of the bacterial 16S rRNA gene, delimited through the primers 341F and 805R, were sequenced in paired-end (2 × 300 cycles) on the Illumina MiSeq platform, according to the Illumina 16S Metagenomic Sequencing Library Preparation protocol.

Afterwards, demultiplexed sequence reads were processed using QIIME2 2022.8. The sequencing primers and the reads without primers were removed using the Cutadapt tool. DADA2 was used to perform paired-end reads filtering, merging, and chimera removal steps after trimming low-quality nucleotides from both forward and reverse reads (--p-trunc-len-f 225 and --p-trunc-len-r 215). Hence, ASVs (Amplicon Sequence Variants) were generated, and the taxonomic assignments were performed through the Scikit-learn multinomial naive Bayes classifier re-trained on the SILVA database (release 138) V3-V4 hyper-variable region. Due to the unavoidable noises which characterize the sequencing of low-microbial-biomass environments, the following measures were taken to minimize sequencing contaminants and improve statistical inferences [13,14]. Every cross-amplified host DNA was identified aligning the ASVs to GRCh38 (human reference genome) using Bowtie2 v.2.2.5. Moreover, the ASVs assigned to Chloroplast or Mitochondria, according to the SILVA database, were removed. Each ASV associated with genera having an average relative abundance under the 0.01% cut-off [15] or found in only one sample across the whole dataset were discarded [16]. Further details about the FASTQ processing are available at https://github.com/LeandroD94/Papers/tree/main/2023_Breast_Cancer_Urban_Rural (accessed on 7 July 2024).

### 2.3. Statistical Analysis

The statistical analyses of bacterial communities were performed in R 4.3 with the help of the packages phyloseq 1.44.0, vegan 2.6–4, DESeq2 1.40.1, and other packages satisfying their dependencies. The packages ggplot2 3.4.2, ggvenn 0.1.9, ggh4x 0.2.4, and ggpubr 0.40 were used to plot the data and results. A saturation analysis on ASVs was performed on every sample using the function rarecurve (step 100 reads), further processed to highlight saturated samples (arbitrarily defined as saturated samples with a final slope in the rarefaction curve with an increment in ASV number per reads < 1 × 10^−5^). The observed richness and Shannon indices were used to estimate the genera α-diversity in each sample using the function estimate_richness from phyloseq. The Pielou’s evenness index was calculated using the formula E = S/log(R), where S is the Shannon diversity index and R is the observed genera richness in the sample. A Venn diagram is used to represent the distribution of the “core” microbiota (here defined as genera with a minimal abundance of 0.1% at least in three samples) among the groups. PCoAs were performed using the Hellinger distance on transformed genera abundances. PERMANOVA and Betadisper were used to test the statistical significance of the β-diversity distances and dispersions, respectively. At different taxonomic ranks, the differential analysis of the abundances has been computed with DESeq2 on raw count data. Furthermore, differentially abundant taxa with a DESeq2 baseMean value < 50 have been discarded from the displayed results, irrespective of their statistical significance to limit noisy results. The resulting *p*-values have been adjusted according to the Benjamini–Hochberg method, and then are considered significative if they are lower than 0.05. Further details about the data analysis are available at https://github.com/LeandroD94/Papers/tree/main/2023_Breast_Cancer_Urban_Rural (accessed on 7 July 2024).

## 3. Results

### 3.1. Patients

In this study, fourteen women with diagnosed BC (mean age of 50.5, range 38–84 years) were enrolled, and their demographic and clinicopathological features are summarized in Table 1. Notably, five patients (35%) had a family history of BC, and the majority (71%) exhibited a Nottingham histologic score of II. Furthermore, most patients (93%) were affected by a non-specific invasive ductal carcinoma and had undergone chemotherapy prior to surgical resection. Finally, half of the patients (50%) lived in urban areas, while the others resided in the rural regions of Algeria.

### 3.2. Evaluation of Breast Tissue-Associated Microbiota in Tumor and Adjacent Healthy Samples

First, we assessed the composition of breast tissue-associated microbiota in both tumor (oncobiome) and adjacent healthy tissue samples.

**Figure 1:**
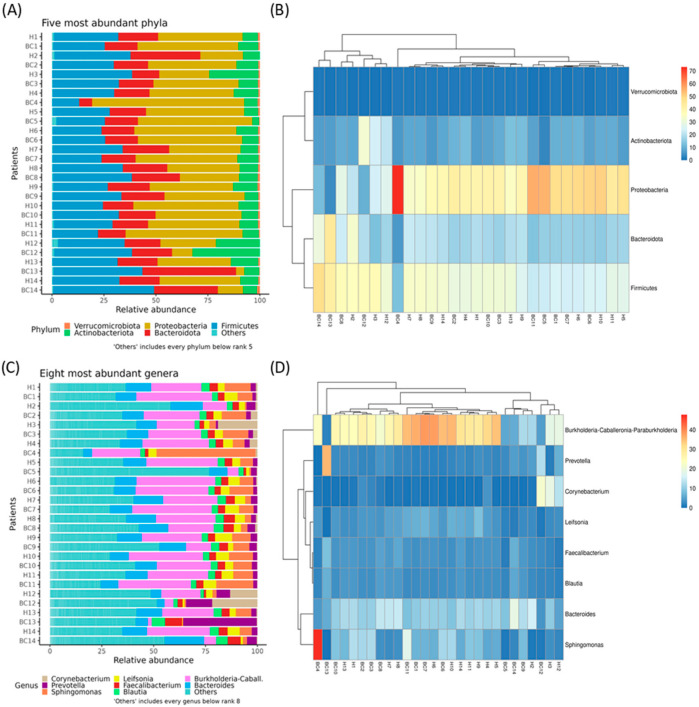
Stacked bar plots showing the relative bacterial abundances of the five most represented phyla (**A**), and a hierarchical clustered heatmap showing the relative abundance of bacterial phyla across different samples (**B**). Stacked bar plots showing relative bacterial abundances of the eight most represented genera (**C**), and a hierarchical clustered heatmap showing the relative abundance of bacterial genera across different samples (**D**).

Across all samples, sequencing yielded an average of 41,720.375 reads per sample (range from 2838 to 140,170) after quality filtering. Detailed read counts for each sample are presented in Table S1. The taxonomic composition of these samples is summarized in Table S2, and the percentage of reads assigned to “unclassified genus” per sample is reported in Table S3. The relative abundances of the five most prevalent phyla and the eight most common genera are shown in Figure 1A–D, respectively. Although no substantial differences were observed between cancerous and healthy tissues, the top five phyla were Proteobacteria (39%), Firmicutes (30%), Bacteroidota (19%), Actinobacteriota (10%), and Verrucomicrobiota (1%). The eight most represented genera were *Bacteroides*, *Blautia*, *Burkholderia*, *Corynebacterium*, *Faecalibacterium*, *Leifsonia*, *Prevotella*, and *Sphingomonas*.

**Figure 2:**
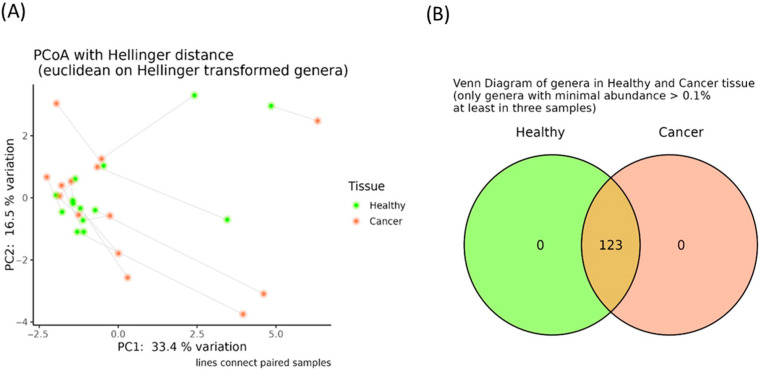
Principal coordinate analysis (PCoA) conducted with the Hellinger distance on the transformed genera abundances of tumor and healthy adjacent breast tissues (**A**). Venn diagram reporting the number of shared genera having a minimal abundance higher than 0.1% and present in at least three samples among healthy (green) and adjacent breast tumor tissues (orange) (**B**).

Additionally, no significant differences in α-diversity indices were identified (Figure S1), and the principal coordinate analysis computed using the Hellinger distance on transformed genera abundances did not reveal distinct clustering between the oncobiome and adjacent healthy tissues (Figure 2A). Finally, as shown in Figure 2B, all genera previously classified as “core microbiota” were present in both cancerous and adjacent tissues. These findings were further supported by the absence of differentially abundant taxa between these tissue types.

### 3.3. Rural–Urban Differences in Oncobiome and Paired Healthy Breast Tissues′ Microbiota

The microbiota composition did not show significant differences between patients stratified according to individual and tumor-related features such as age, chemo/radiotherapy, hormonal therapy, cancer grade, and size.

**Figure 3:**
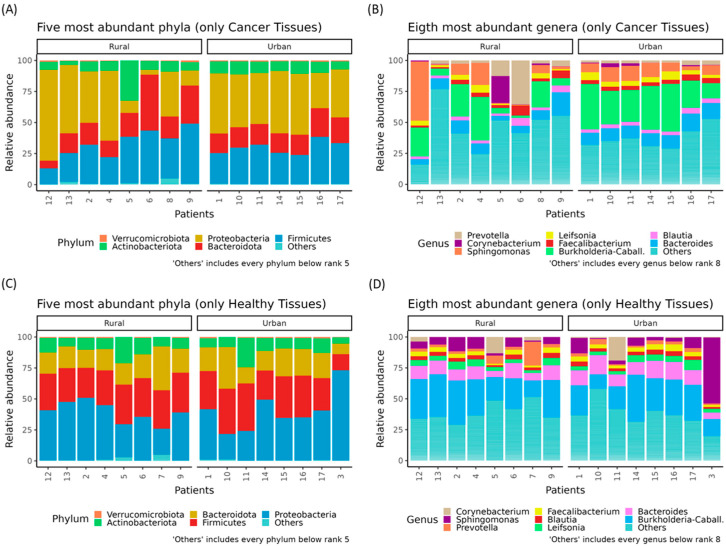
Relative abundances (%) of the top phyla and genera in BC patients, stratified by residential area. Stacked bar plots showing the five most abundant phyla in cancer tissues of BC patients from urban or rural areas (**A**). Stacked bar plots showing the eight most abundant genera in cancer tissues of BC patients from urban or rural areas (**B**). Stacked bar plots showing the five most abundant phyla in healthy tissues of BC patients from urban or rural areas (**C**). Stacked bar plots showing the eight most abundant genera in healthy tissues of BC patients from urban or rural areas (**D**).

However, an interesting variation was observed when comparing patients from different residential areas. The microbiota composition differed between patients from rural and urban settings, with notable variations in the relative abundance of key phyla and genera (Figure 3).

**Figure 4:**
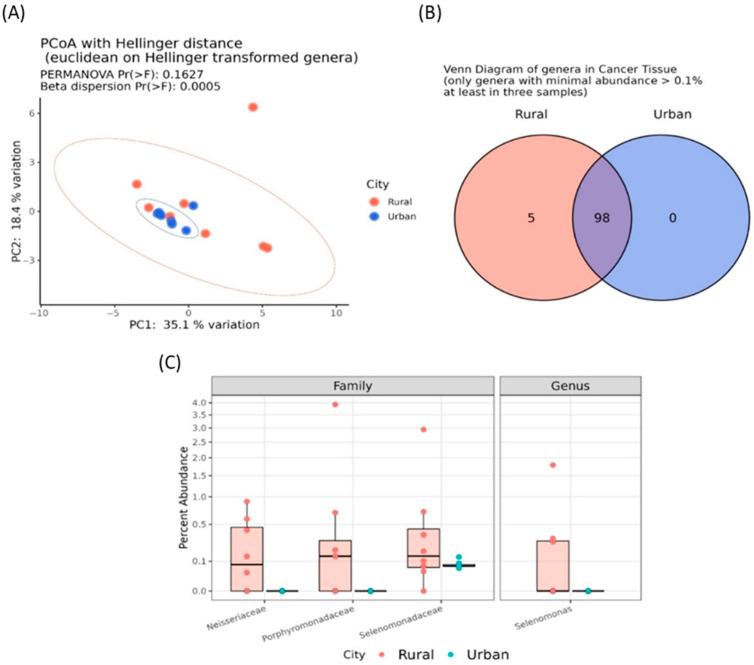
Principal coordinate analysis (PCoA) conducted with the Hellinger distance on transformed genera abundances of cancer tissues of BC patients from urban or rural areas (**A**). Venn diagram showing the number of shared genera having a minimal abundance higher than 0.1% and present at least in three samples among cancer tissues of BC patients from rural (red) or urban areas (blue) (**B**). Boxplots reporting the significant differentially abundant taxa among cancer tissues of BC patients from rural or urban areas (**C**).

In addition, although no significant α-diversity indices were reported (Figure S3), a significant β dispersion (*p* < 0.0005) was detected among tumor samples from patients living in urban versus rural areas (Figure 4A). Additionally, five genera of the so-called “core microbiota”, namely *Selenomonas*, *Centipeda*, *Leptotrichia*, *Neisseria*, and *Porphyromonas*, were exclusively present in the oncobiome of patients from rural areas (Figure 4B).

Patients from rural areas also exhibited a significant increase in bacteria belonging to the Neisseriaceae (log2FC = 9.562; padj < 0.0001), Porphyromonadaceae (log2FC = 10.696; padj < 0.0001), and Selenomonadaceae (log2FC = 3.558; *p* adj = 0.003) families, as well as the *Selenomonas* genus (log2FC = 25.447; padj < 0.0001) (Figure 4C). Remarkably, no significant microbiota differences were found between tumor-adjacent healthy tissues of patients from urban and rural areas, further reinforcing these findings (Figure S4).

### 3.4. PICRUST Analysis

**Figure 5:**
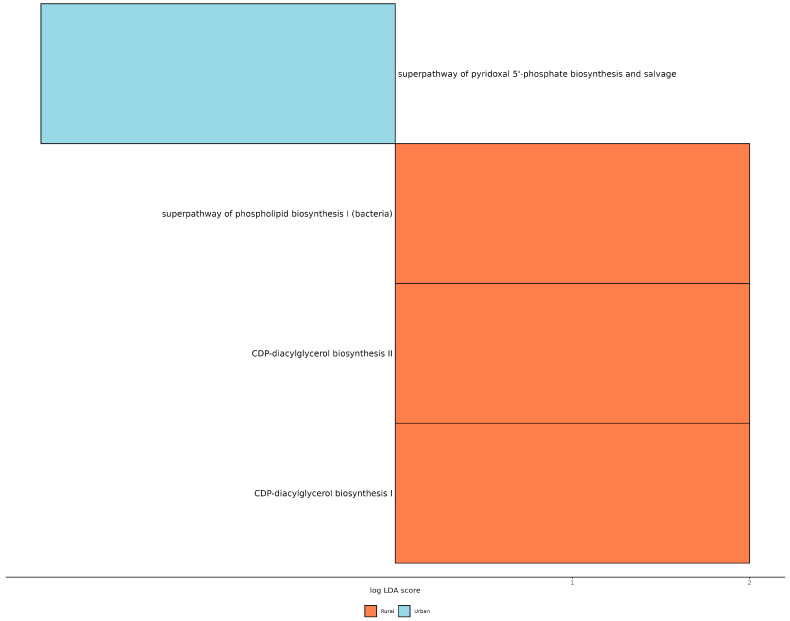
Statistically significant different predicted pathway with LDA score > 2.0 between BC patients from urban and rural areas.

Finally, we applied the PICRUSt2 (phylogenetic investigation of communities by reconstruction of unobserved states) predictive metabolism approach on the 16S rRNA gene sequencing data to assess functional and metabolic changes within the mammary microbial communities. The results reveal a significant increase in the mixed acid fermentation pathway in tumor tissues (*p* = 0.022), while the superpathway of glucose and xylose degradation was significantly elevated (*p* = 0.032) in the healthy tissues of BC patients (Figure S5). Interestingly, when comparing patients from different living areas, BC patients from rural areas exhibited enhanced pathways related to CDP-diacylglycerol biosynthesis I (*p* = 0.039), CDP-diacylglycerol biosynthesis II (*p* = 0.039), and phospholipid biosynthesis I (*p* = 0.047) (Figure 5).

## 4. Discussion

BC incidence has reached unprecedented levels globally in recent decades, but the underlying causes for most cases remain unknown. However, emerging research suggests that the mammary microbiota differs between BC patients and healthy individuals, indicating that specific bacterial communities may contribute to cancer development [17]. In addition to known risk factors such as diet, gastrointestinal disorders, hormonal imbalances, and immune system dysfunctions, the living environment is now recognized as a critical factor influencing microbial imbalances and potentially promoting BC onset and progression [18].

Analysis of published bioinformatics datasets has revealed variations in microbiota composition among BC patients of different ethnicities, as well differences between benign and malignant BC tissues and across BC subtypes [19].

In detail, breast tissues of Algerian patients were characterized by the presence, in descending order, of Proteobacteria, Firmicutes, Bacteroidota, Actinobacteriota, and Verrucomicrobiota phyla. At the genus level, *Bacteroides*, *Blautia*, *Burkholderia*, *Corynebacterium*, *Faecalibacterium*, *Leifsonia*, *Prevotella*, and *Sphingomonas* were the most abundant. Overall, our results document no significant differences in the composition or abundance of bacterial species between cancerous and adjacent healthy tissues.

Similarly, in a recent study of Italian BC patients, no significant differences were observed between tumor and non-pathological adjacent tissues in terms of microbial community structure or bacterial taxa composition [10]. These findings suggest that microbial dysbiosis may extend throughout the entire breast tissue, a trend observed in both Italian and Algerian cohorts and in line with our results. Regarding correlations between microbial composition and clinical parameters, although our BC cohort exhibited a range of Nottingham histologic score, reflecting the inherent heterogeneity of tumor pathology, and all patients had received chemotherapy prior to surgery, we did not identify any clear associations. A recent investigation into gut microbiome dynamics in BC patients demonstrated that chemotherapy can induce significant alterations in microbial α-diversity, with both treatment modality and timing influencing these changes [20]. Consequently, because our study focuses on biopsy rather than fecal microbiota, we cannot exclude the possibility that our tissue-associated microbial profiles represent a post-chemotherapy state. Future studies should, therefore, include both pre- and post-treatment sampling to stratify chemotherapy’s impact on the tissue microbiome.

However, notably, significant differences in microbial composition and abundance were detected only in the oncobiome of patients from different geographical settings (rural vs. urban), not in surrounding healthy tissues. This aligns with expectations, as lifestyle and dietary habits are known to shape microbiome profiles, influencing their diversity and composition in the different human niches, including the breast.

Numerous studies have reported variations in fecal microbiota composition between rural and urban populations [21], with rural environments typically associated with higher microbial diversity, often indicative of a healthier gut microbiome [22]. Since diet and environmental factors significantly influence gut microbiota composition, their effects likely extend to the breast microbiota as well. The colonization of breast tissue occurs primarily through two mechanisms: intestinal translocation, where microbes migrate from the gut, and direct contact during lactation, skin–nipple interaction, and sexual contact [23]. Our findings raise relevant questions about the potential role of environmental factors in breast carcinogenesis within different populations. Additionally, they prompt an exploration of whether these microbial differences can be modulated and what factors might drive such modulation. In detail, we identified that the genera *Selenomonas*, *Centipeda*, *Leptotrichia*, *Neisseria*, and *Porphyromonas* were exclusively present in cancerous tissues of patients from rural areas, but were absent in those from urban settings. Notably, certain periodontal pathogens, such as *Fusobacterium* spp., *Porphyromonas* spp., and *Campylobacter* spp., commonly associated with gastrointestinal infections, belong to a “mobile microbiome”. These bacteria originate in the oral cavity, but can translocate to other human body sites, causing systemic infections and inflammation [24]. Furthermore, while *Streptococcus* spp., *Neisseria* spp., and *Veillonella* spp. are associated with anti-inflammatory processes, *Selenomonas* spp., *Parvimonas* spp., and *Campylobacter* spp. are linked to pro-inflammatory conditions [25]. Interestingly, *Selenomonas* spp. has been found to be overabundant in saliva and tumor tissues of lung cancer patients, as well as in several other cancer types [26,27]. Meanwhile, *Leptotrichia* spp., *Porphyromonas* spp., and *Selenomonas* spp. are known oral bacteria associated with halitosis, a condition affecting 15–60% of the population. The bacterial production of volatile compounds like amines, alcohols, aldehydes, and ketones, can be toxic to human cells, even at low concentrations, potentially explaining their carcinogenic role [28].

In summary, our findings highlight the presence of potential driver and passenger bacteria in BC patients from rural areas, which may influence risk and progression of BC. This is in line with the study by Hussein and colleagues, who observed that a significant proportion of BC patients were from rural regions, whereas most women with benign breast lesions resided in urban areas [26]. Although it remains unclear whether these observed regional disparities are a cause of cancer or merely a consequence, they underscore the impact of environmental and lifestyle factors on microbial community composition and cancer susceptibility.

Microbiome alterations can impact cancer risk both at the primary site and in distant tissues through mechanisms such as inflammation promotion, modulation of the tissue microenvironment, metabolic changes, and direct cytotoxic effects [29]. This evidence suggests that bacteria play a role in maintaining breast tissue homeostasis by regulating host inflammatory responses. A decrease in bacterial load in healthy individuals may, therefore, exacerbate BC risk. Indeed, the mammary microbiota may influence BC development and progression not only by modulating local estrogen levels, but also by shaping inflammatory responses and immune trafficking within the tumor microenvironment [30].

Lastly, functional metagenomics analysis using PICRUSt2 revealed increased phospholipid biosynthesis, particularly through the CDP-diacylglycerol biosynthesis I (*p* = 0.039), CDP-diacylglycerol biosynthesis II (*p* = 0.039), and phospholipid biosynthesis I (*p* = 0.047) pathways, in BC patients from rural areas compared to those from urban areas. Elevated phospholipid biosynthesis in BC plays a crucial role in processes such as cell proliferation, survival, and metastasis. Lipid profiling of BC tissue samples has demonstrated a higher phospholipid content compared to adjacent non-cancerous breast tissues [30]. Moreover, the concentrations of phosphatidylcholine and phosphatidylethanolamine have been observed to increase with BC tumor grade, suggesting that phospholipid synthesis rates escalate with oncogenesis and tumor progression relative to normal tissue [31].

Despite its strengths, this study shows some limitations. First, this analysis was conducted on a relatively small number of patients. This limitation arose from our decision to work with fresh samples, thereby avoiding paraffin embedding, which is known to introduce unpredictable microbial contaminants. Nonetheless, all statistical analyses yielded robust and convincing results. Second, as is well-documented in the literature, DNA extraction from low-microbial-biomass environments is inherently challenging, often resulting in off-target amplification from host DNA. After filtering out these sequences, we observed a reduction in overall microbial abundance data, which was expected given the nature of the samples. Lastly, while stringent precautions were taken to maintain sterility throughout sample processing, confirmed by the absence of PCR signals in negative controls, we cannot entirely rule out the presence of trace contaminants. This is a common challenge in microbiome studies, as contaminants are known to be ubiquitous in DNA extraction kits and PCR reagents.

## 5. Conclusions

Our findings document the presence of distinct compositional and functional breast oncobiome profiles between BC patients from rural and urban areas, suggesting that environmental factors influence microbiota composition leading to the development of BC. Surely, considering that confounding factors, such as limited screening programs and the higher prevalence of late-stage diagnoses, are well-known challenges in Algeria, future comparative studies involving populations from diverse cultural and healthcare backgrounds would further enhance the generalizability and impact of our findings, as well as their implications for BC prevention and treatment.

## Figures and Tables

**Table 1:** Demographic and clinical features of enrolled patients. U: urban; R: rural; invasive ductal carcinoma (IDC), invasive lobular carcinoma (ILC).

Patient	Age	Weight (kg)	Area	Family Background	Cancer Type	Cancer Grade	Radio/Chemotherapy
1	≥50	50	U	NO	IDC	Nottingham type II	Chemotherapy
	50>	64	R	YES	IDC	Nottingham type III	Chemotherapy
3	≥50	56	R	NO	IDC	Nottingham II	Chemotherapy
4	50>	72	R	NO	IDC	Nottingham type II	Chemotherapy
5	50>	76	R	NO	IDC	Nottingham II	Chemotherapy
6	50>	72	R	NO	IDC	Nottingham type II	Chemotherapy
7	≥50	76	U	NO	IDC	Nottingham type III	Chemotherapy
8	≥50	55	U	NO	IDC	Nottingham type I	Chemotherapy
9	≥50	70	R	NO	ILC	Nottingham type II	Radiotherapy
10	50>	60	R	YES	IDC	Nottingham type II	Chemotherapy
11	50>	80	U	NO	IDC	Nottingham type II	Chemotherapy
12	≥50	70	U	YES	IDC	Nottingham type II	Chemotherapy
13	50>	72	U	YES	IDC	Nottingham type III	Chemotherapy
14	50>	74	U	YES	IDC	Nottingham type II	Chemotherapy

## Data Availability

The data generated in the present study may be found in the Gene Expression Omnibus (GEO) repository under accession number (GSE243440).

## Supplementary Materials

The following supporting information can be downloaded at: https://www.mdpi.com/article/10.3390/genes16070806/s1, Figure S1: Box plots showing α-diversity indices (Observed ASV, Shannon index, Pielou’s evenness) among tumor and healthy adjacent breast tissues; Figure S2: Hierarchical clustered heatmap showing the relative abundance of the five most abundant bacterial phyla (A) and the eight most abundant genera (B) in cancer tissues of BC patients from urban or rural areas. Hierarchical clustered heatmap showing the relative abundance of the five most abundant bacterial phyla (C) and the eight most abundant genera (D) in healthy tissues of BC patients from urban or rural areas; Figure S3: Box plots showing α-diversity indices (Observed ASV, Shannon index, Pielou’s evenness) among tumor and healthy adjacent breast tissues of patients living in urban (A) or in rural areas (B); Figure S4: Principal coordinate analysis (PCoA) conducted with the Hellinger distance on transformed genera abundances of tumor-surrounding healthy tissues of BC patients from urban or rural areas (A). Venn diagram showing the number of shared genera having a minimal abundance higher than 0.1% among tumor-surrounding healthy tissues of BC patients from urban or rural areas (B); Figure S5: Statistically significant different predicted pathway with LDA score > 2.0 between mammary healthy and adjacent tumor tissues of BC patients; Table S1: Read counts for each sample after quality filtering. BC: breast cancer; Table S2: Summary of the taxonomic analysis of the obtained ASVs from breast tissues of BC patients. ASV: amplicon sequence variants, BC: breast cancer; Table S3: Percentage of reads assigned to “unclassified genus” per sample. BC: breast cancer.

## References

[1] Sung H., Ferlay J., Siegel R.L., Laversanne M., Soerjomataram I., Jemal A., Bray F. (2021). Global Cancer Statistics 2020: GLOBOCAN Estimates of Incidence and Mortality Worldwide for 36 Cancers in 185 Countries. CA Cancer J. Clin..

[2] Thun M., Linet M.S., Cerhan J.R., Haiman C.A., Schottenfeld D. (2017). Cancer Epidemiology and Prevention.

[3] Najjar H., Easson A. (2010). Age at diagnosis of breast cancer in Arab nations. Int. J. Surg..

[4] Uhrhammer N., Abdelouahab A., Lafarge L., Feillel V., Ben Dib A., Bignon Y.J. (2008). BRCA1 mutations in Algerian breast cancer patients: High frequency in young, sporadic cases. Int. J. Med. Sci..

[5] Corbex M., Bouzbid S., Boffetta P. (2014). Features of breast cancer in developing countries, examples from North-Africa. Eur. J. Cancer.

[6] Smaili F., Boudjella A., Dib A., Braikia S., Zidane H., Reggad R., Bendib A., Abdelouahab A., Bereksi-Reguig F., Yekrou D. (2020). Epidemiology of breast cancer in women based on diagnosis data from oncologists and senologists in Algeria. Cancer Treat. Res. Commun..

[7] Bernardo G., Le Noci V., Di Modica M., Montanari E., Triulzi T., Pupa S.M., Tagliabue E., Sommariva M., Sfondrini L. (2023). The Emerging Role of the Microbiota in Breast Cancer Progression. Cells.

[8] German R., Marino N., Hemmerich C., Podicheti R., Rusch D.B., Stiemsma L.T., Gao H., Xuei X., Rockey P., Storniolo A.M. (2023). Exploring breast tissue microbial composition and the association with breast cancer risk factors. Breast Cancer Res. BCR.

[9] Esposito M.V., Fosso B., Nunziato M., Casaburi G., D’Argenio V., Calabrese A., D’Aiuto M., Botti G., Pesole G., Salvatore F. (2022). Microbiome composition indicate dysbiosis and lower richness in tumor breast tissues compared to healthy adjacent paired tissue, within the same women. BMC Cancer.

[10] Thompson K.J., Ingle J.N., Tang X., Chia N., Jeraldo P.R., Walther-Antonio M.R., Kandimalla K.K., Johnson S., Yao J.Z., Harrington S.C. (2017). A comprehensive analysis of breast cancer microbiota and host gene expression. PLoS ONE.

[11] Niccolai E., Baldi S., Nannini G., Gensini F., Papi L., Vezzosi V., Bianchi S., Orzalesi L., Ramazzotti M., Amedei A. (2023). Breast cancer: The first comparative evaluation of oncobiome composition between males and females. Biol. Sex Differ..

[12] Xuan C., Shamonki J.M., Chung A., Dinome M.L., Chung M., Sieling P.A., Lee D.J. (2014). Microbial dysbiosis is associated with human breast cancer. PLoS ONE.

[13] Allali I., Delgado S., Marron P.I., Astudillo A., Yeh J.J., Ghazal H., Amzazi S., Keku T., Azcarate-Peril M.A. (2015). Gut microbiome compositional and functional differences between tumor and non-tumor adjacent tissues from cohorts from the US and Spain. Gut Microbes.

[14] Cao Q., Sun X., Rajesh K., Chalasani N., Gelow K., Katz B., Shah V.H., Sanyal A.J., Smirnova E. (2020). Effects of Rare Microbiome Taxa Filtering on Statistical Analysis. Front. Microbiol..

[15] Walker S.P., Tangney M., Claesson M.J. (2020). Sequence-Based Characterization of Intratumoral Bacteria-A Guide to Best Practice. Front. Oncol..

[16] Karstens L., Asquith M., Davin S., Fair D., Gregory W.T., Wolfe A.J., Braun J., McWeeney S., Gilbert J.A. (2019). Controlling for Contaminants in Low-Biomass 16S rRNA Gene Sequencing Experiments. mSystems.

[17] Nearing J.T., Douglas G.M., Hayes M.G., MacDonald J., Desai D.K., Allward N., Jones C.M.A., Wright R.J., Dhanani A.S., Comeau A.M. (2022). Microbiome differential abundance methods produce different results across 38 datasets. Nat. Commun..

[18] Fernández M.F., Reina-Pérez I., Astorga J.M., Rodríguez-Carrillo A., Plaza-Díaz J., Fontana L. (2018). Breast Cancer and Its Relationship with the Microbiota. Int. J. Environ. Res. Public Health.

[19] Bodai B.I., Nakata T.E. (2020). Breast Cancer: Lifestyle, the Human Gut Microbiota/Microbiome, and Survivorship. Perm. J..

[20] Wu A.H., Vigen C., Tseng C., Garcia A.A., Spicer D. (2022). Effect of Chemotherapy on the Gut Microbiome of Breast Cancer Patients During the First Year of Treatment. Breast Cancer.

[21] Parida S., Sharma D. (2019). The power of small changes: Comprehensive analyses of microbial dysbiosis in breast cancer. Biochim. Biophys. Acta Rev. Cancer.

[22] Tamburini F.B., Maghini D., Oduaran O.H., Brewster R., Hulley M.R., Sahibdeen V., Norris S.A., Tollman S., Kahn K., Wagner R.G. (2022). Short- and long-read metagenomics of urban and rural South African gut microbiomes reveal a transitional composition and undescribed taxa. Nat. Commun..

[23] De Filippo C., Di Paola M., Ramazzotti M., Albanese D., Pieraccini G., Banci E., Miglietta F., Cavalieri D., Lionetti P. (2017). Diet, Environments, and Gut Microbiota. A Preliminary Investigation in Children Living in Rural and Urban Burkina Faso and Italy. Front. Microbiol..

[24] Gomez-Gallego C., Garcia-Mantrana I., Salminen S., Collado M.C. (2016). The human milk microbiome and factors influencing its composition and activity. Semin. Fetal Neonatal Med..

[25] Mukherjee P.K., Wang H., Retuerto M., Zhang H., Burkey B., Ghannoum M.A., Eng C. (2017). Bacteriome and mycobiome associations in oral tongue cancer. Oncotarget.

[26] Joshi V., Matthews C., Aspiras M., de Jager M., Ward M., Kumar P. (2014). Smoking decreases structural and functional resilience in the subgingival ecosystem. J. Clin. Periodontol..

[27] Yan X., Yang M., Liu J., Gao R., Hu J., Li J., Zhang L., Shi Y., Guo H., Cheng J. (2015). Discovery and validation of potential bacterial biomarkers for lung cancer. Am. J. Cancer Res..

[28] Rodriguez R.M., Hernandez B.Y., Menor M., Deng Y., Khadka V.S. (2020). The landscape of bacterial presence in tumor and adjacent normal tissue across 9 major cancer types using TCGA exome sequencing. Comput. Struct. Biotechnol. J..

[29] Hampelska K., Jaworska M.M., Babalska Z., Karpiński T.M. (2020). The Role of Oral Microbiota in Intra-Oral Halitosis. J. Clin. Med..

[30] Khan A.A., Sirsat A.T., Singh H., Cash P. (2022). Microbiota and cancer: Current understanding and mechanistic implications. Clin. Transl. Oncol..

[31] Mikó E., Kovács T., Sebő É., Tóth J., Csonka T., Ujlaki G., Sipos A., Szabó J., Méhes G., Bai P. (2019). Microbiome-Microbial Metabolome-Cancer Cell Interactions in Breast Cancer-Familiar, but Unexplored. Cells.

