# Supplementary material for: Compositional and Functional Disparities in the Breast Oncobiome Between Patients Living in Urban or Rural Areas
Source: Genes (Basel). 2025 Jul 9;16(7):806. doi: 10.3390/genes16070806 (PMC12295151; doi:10.3390/genes16070806)
Supplement: Supplementary file 1 [file genes-16-00806-s001.zip › genes-3728231-supplementary.pdf]

SUPPLEMENTARY MATERIALS

Figure S1. Box plots showing alpha diversity indices (Observed ASV, Shannon index, Pielou's evenness) among tumor and healthy adjacent breast tissues.

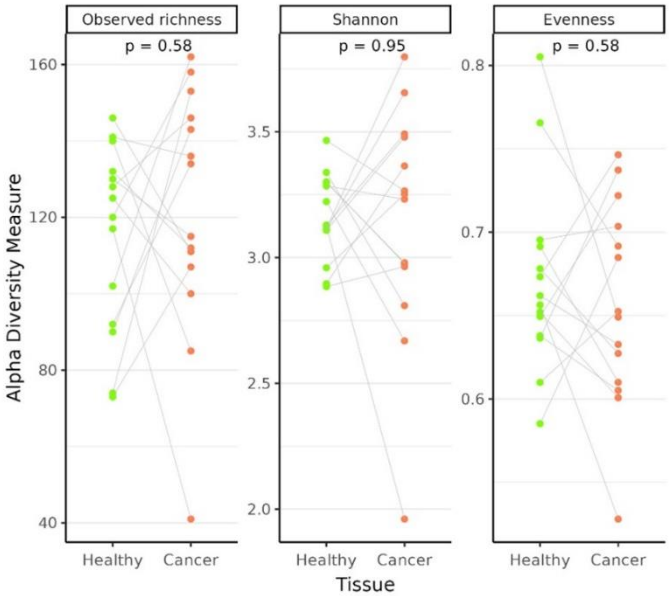

Figure S2. Hierarchical clustered heatmap showing the relative abundance of the five most abundant bacterial phyla (A) and the eight most abundant genera (B) in cancer tissues of BC patients from urban or rural areas. Hierarchical clustered heatmap showing the relative abundance of the five most abundant bacterial phyla (C) and the eight most abundant genera (D) in healthy tissues of BC patients from urban or rural areas.

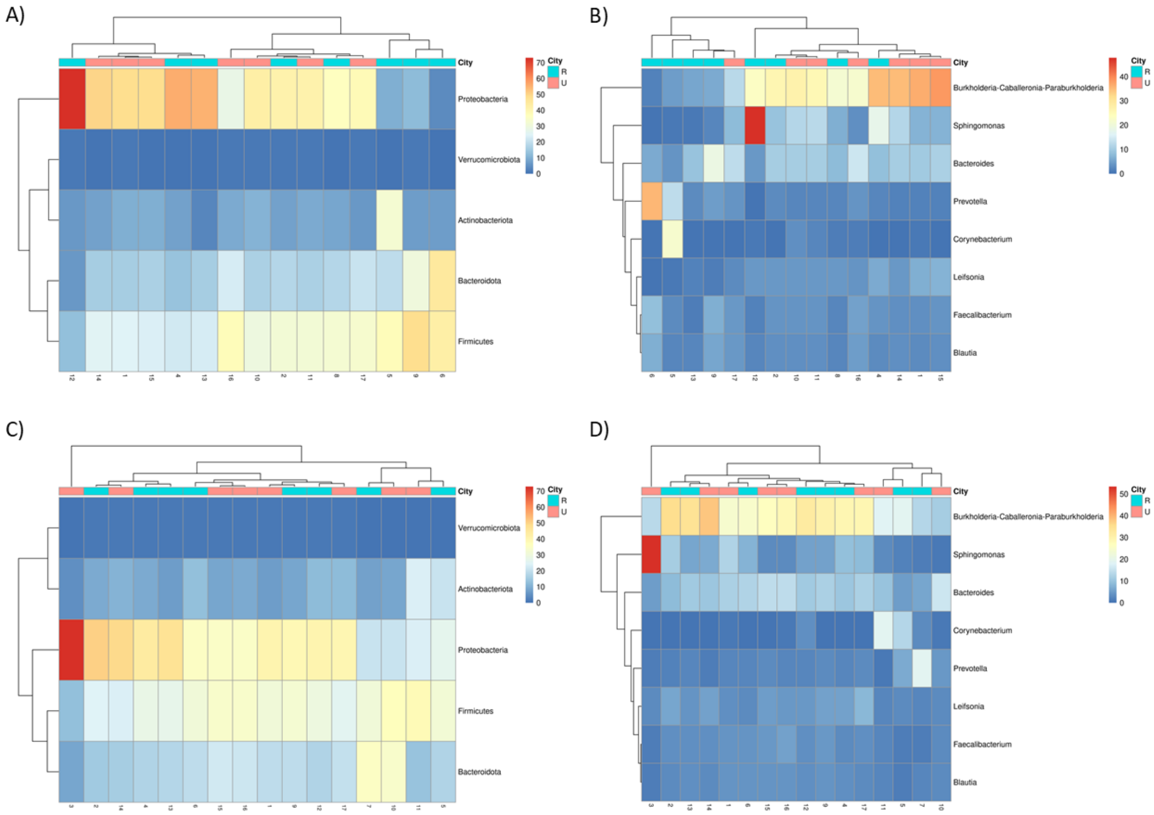

Figure S3. Box plots showing alpha diversity indices (Observed ASV, Shannon index, Pielou's evenness) among tumor and healthy adjacent breast tissues of patients living in urban (A) or in rural areas (B).

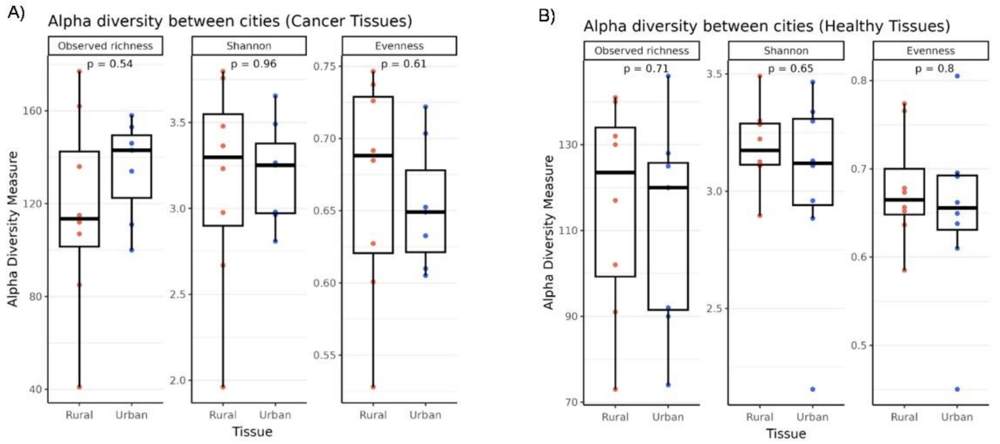

Figure S4. Principal coordinate analysis (PCoA) conducted with the Hellinger distance on transformed genera abundances of tumor-surrounding healthy tissues of BC patients from urban or rural areas (A). Venn diagram showing the number of shared genera having a minimal abundance higher than 0.1% among tumor-surrounding healthy tissues of BC patients from urban or rural areas (B).

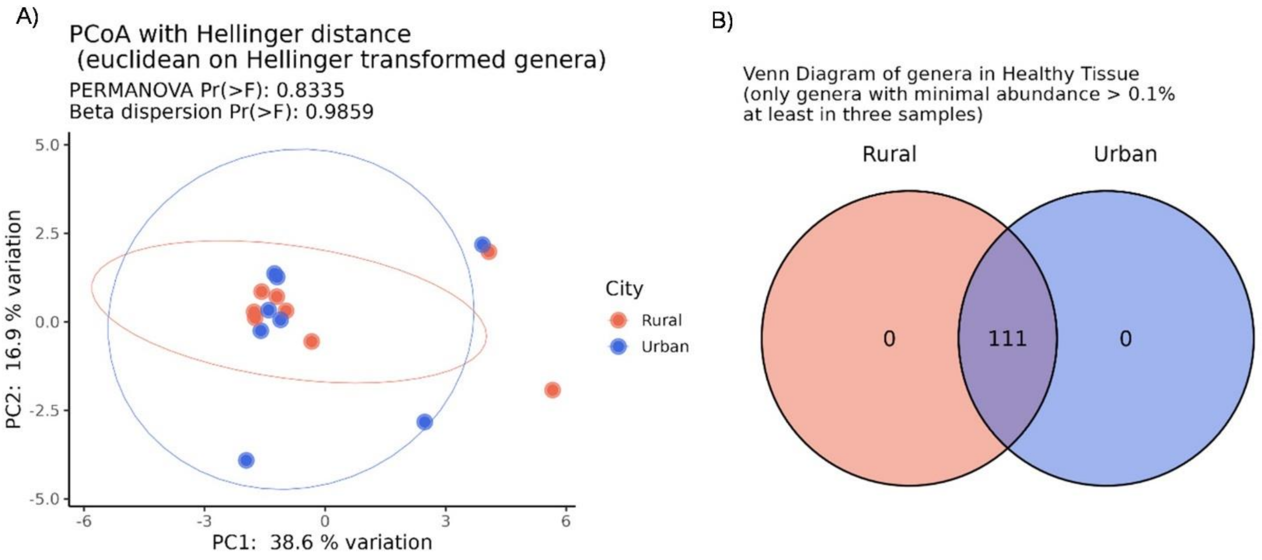

Figure S5. Statistically significant different predicted pathway with LDA score > 2.0 between mammary healthy and adjacent tumor tissues of BC patients.

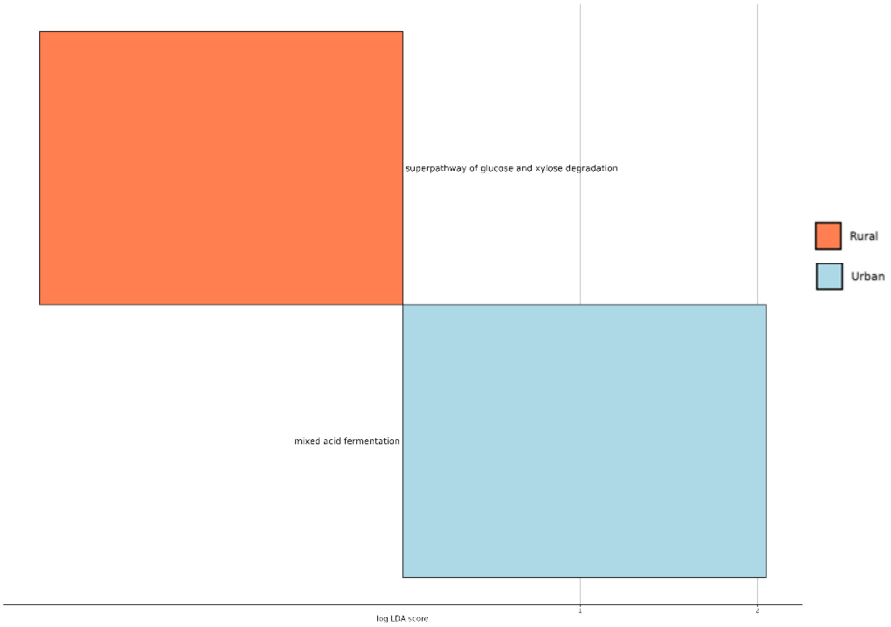

Table S1. Read counts for each sample after quality filtering. BC: breast cancer

SampleID	Filtered sequences
BC1	15395
BC2	56003
BC3	75427
BC4	2838
BC5	18183
BC6	36719
BC7	15213
BC8	42943
BC9	140170
BC10	29948
BC11	9929
BC12	15035
BC13	33234
BC14	93404
H1	53002
H2	4660
H3	65957
H4	25426
H5	37181
H6	11767
H7	26990
H8	8723
H9	29891
H10	73583
H11	54169
H12	18221
H13	34621
H14	3768

Table S2. Summary of the taxonomic analysis of the obtained ASVs from breast tissues of BC patients. ASV: amplicon sequence variants, BC: breast cancer

Rank	Total ASV	Assigned ASV	% ASV
Phylum	17	17	100
Class	29	28	96.55
Order	70	66	94.29
Family	121	109	90.08
Genus	264	225	85.23

Table S3. Percentage of reads assigned to "unclassified genus" per sample. BC: breast cancer

SampleID	% Unassigned Genera
BC1	1.29
BC2	1.30
BC3	1.23
BC4	0.00
BC5	22.63
BC6	1.55
BC7	1.17
BC8	1.63
BC9	8.18
BC10	1.90
BC11	1.95
BC12	1.46
BC13	1.02
BC14	1.66
H1	1.26
H2	1.42
H3	0.85
H4	1.8
H5	2.58
H6	1.09
H7	1.75
H8	0.83
H9	1.25
H10	1.39
H11	1.15
H12	1.59
H13	1.39
H14	1.22
